# Supplementary material for: Dense time-course gene expression profiling of the Drosophila melanogaster innate immune response
Source: BMC Genomics. 2021 Apr 26;22:304. doi: 10.1186/s12864-021-07593-3 (PMC8074482; doi:10.1186/s12864-021-07593-3)
Supplement: Supplementary file 1 — Additional file 1 Figure S1. Plots of normalized counts of housekeeping genes and immune response genes. Figure S2. Venn Diagram showing overlap and differences of DE genes identified using limma-voom spline fitting vs. maSigPro fitting of polynomials. Figure S3. Heatmap of 214 genes. Figure S4. Temporal dynamics of gene expression of the most strongly up-regulated genes . Figure S5. Expression profiles of DE genes encoding transcription factors. Figure S6. Gluconeogenesis pathway. Figure S7. GC filtered network of negative edges. Figure S8. Negative GC edges. Figure S9. Pathway corresponding to ‘mitotic DNA replication checkpoint’. Figure S10. GC edges of circadian rhythm genes plotted against time. Figure S11. Positive GC edges. Figure S12. Outlier explanation [file 12864_2021_7593_MOESM1_ESM.docx]

## SUPPLEMENTAL FIGURES


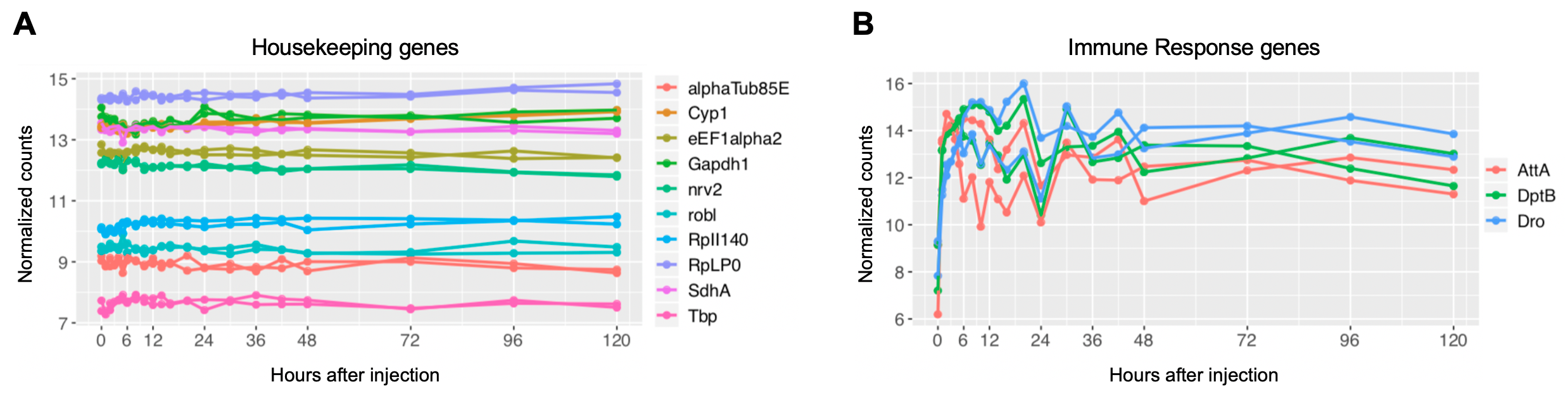


**Figure S1**. Plots of normalized counts of housekeeping genes (**A**) show little change across time as expected under proper data normalization, while immune response genes (**B**) show up-regulation within the first time points, as expected after a successful Imd stimulation.

**
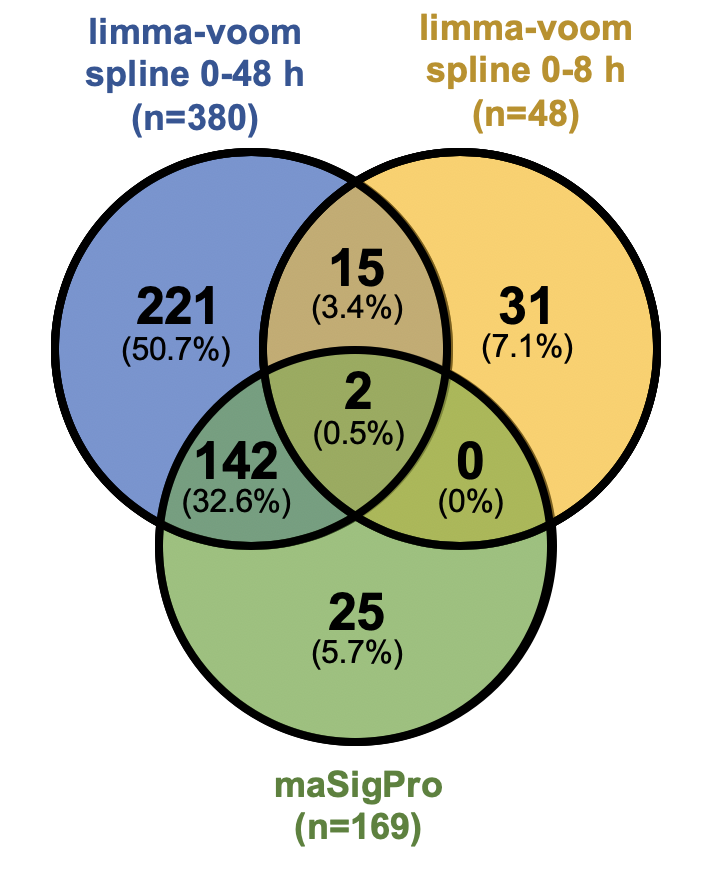
**

**Figure S2.** Venn Diagram showing overlap and differences of DE genes identified using limma-voom spline fitting vs. maSigPro fitting of polynomials.


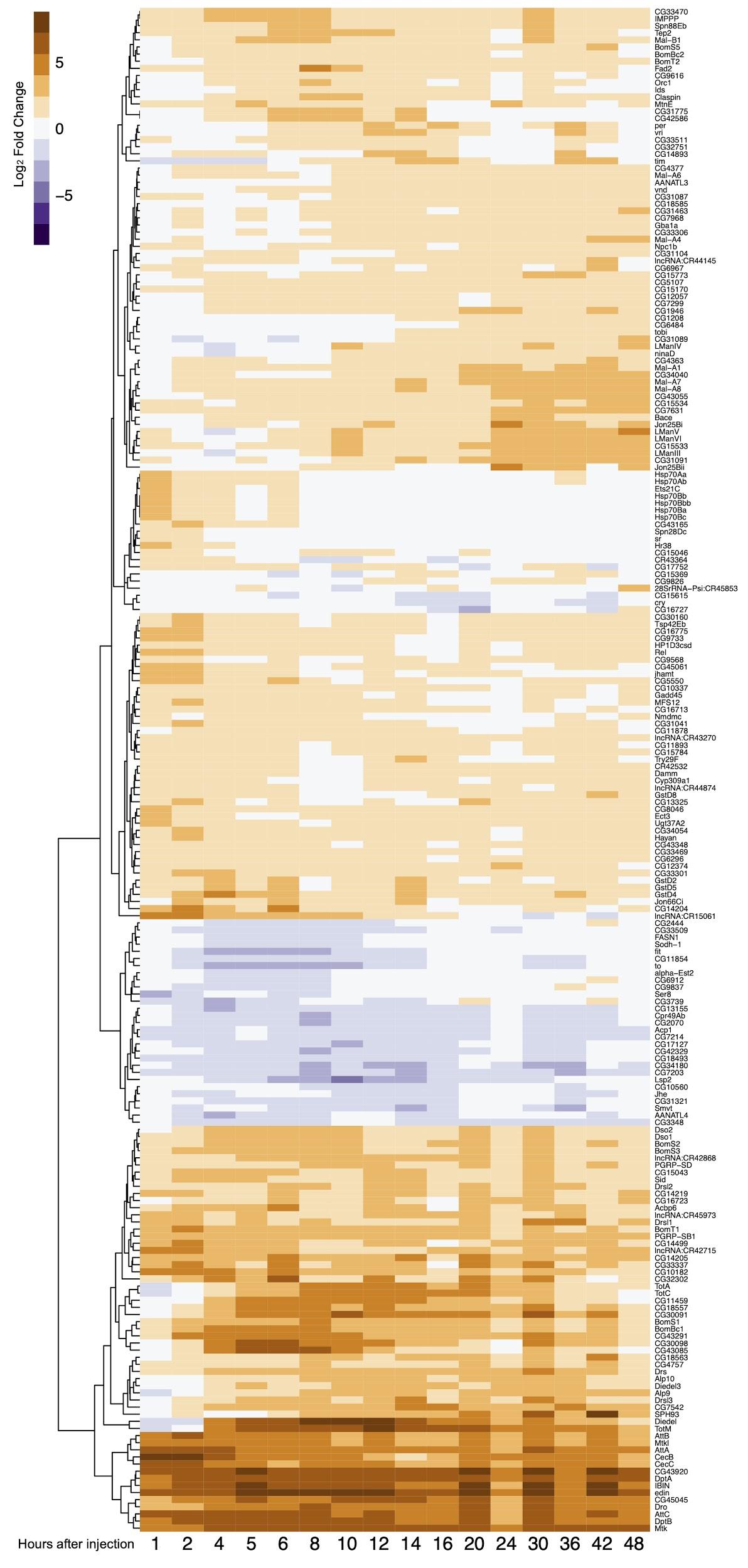


**Figure S3. Heatmap of 214 genes differentially expressed following injection with commercial LPS.** Scale is log_2_ fold change, with negative expression in purple and positive expression in dark orange, compared to baseline.

**
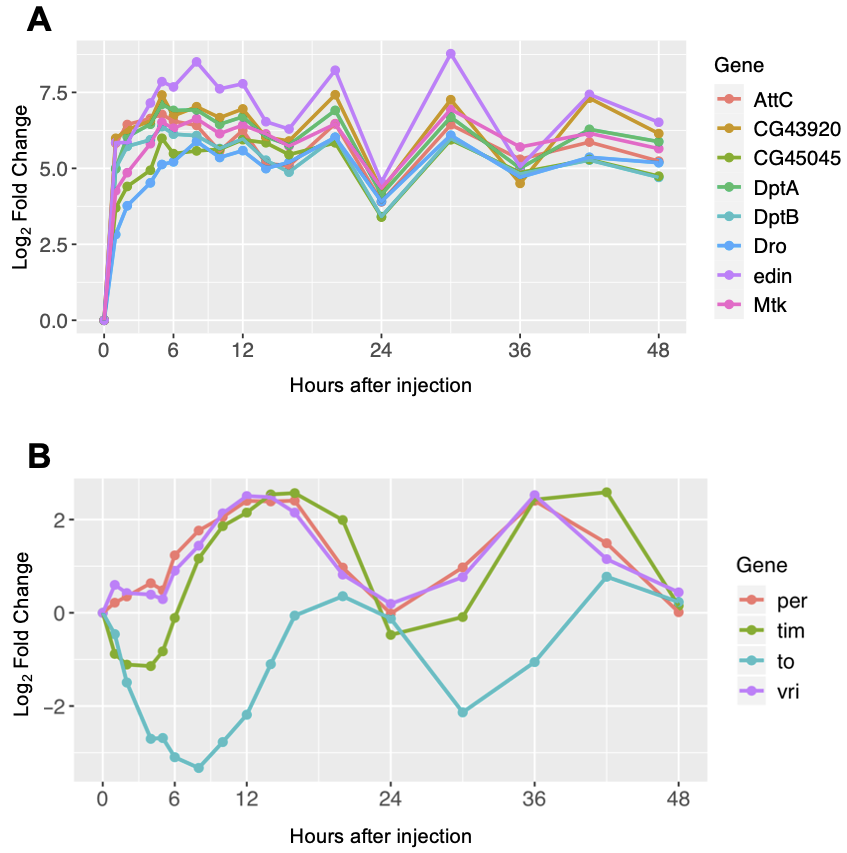
**

**Figure S4. (A)** Temporal dynamics of the most strongly up-regulated genes (*DptB*, *AttC*, *Mtk*, *Dro*, *CG45045*, *DptA*, *CG43920*, and *edin*) in the first 48 hr after injection. These genes are part of the 91 “core” DE genes with log_2_FC > 2 in at least two time intervals after injection, with an FDR < 0.01 (**Figure 3**). (**B**) Temporal dynamics of gene expression of circadian rhythm genes (*per*, *tim*, *to*, *vri*) show a classic and well characterized 24 hr periodic expression.

**
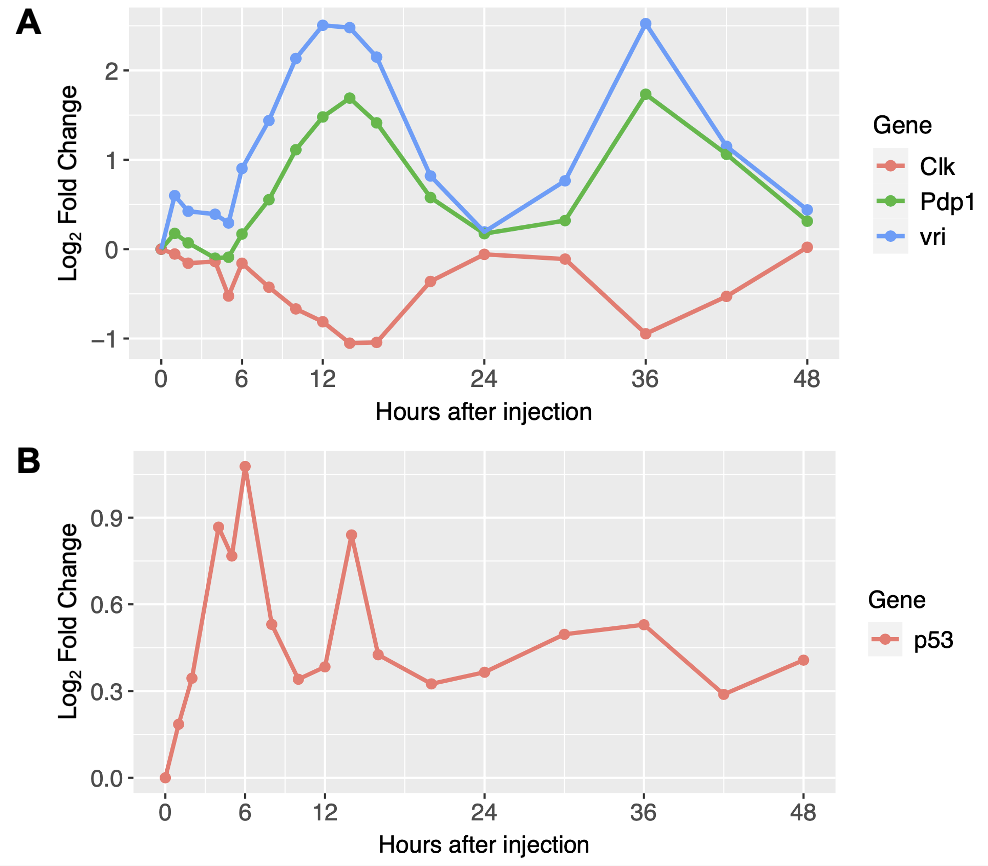
**

**Figure S5. Expression profiles of DE genes encoding transcription factors. (A)** Genes involved in regulation of the circadian clock. **(B)** *p53***,** which encodes a transcription factor involved in DNA repair and genotoxic stress.

**
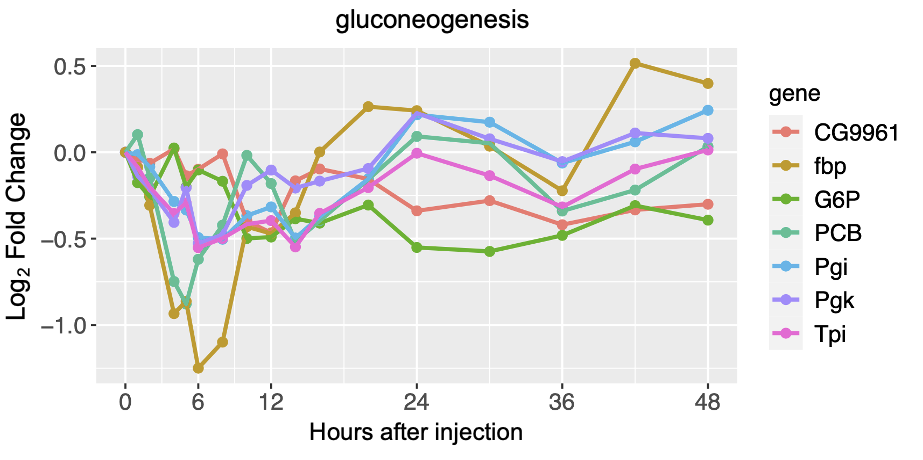
**

**Figure S6.** Gluconeogenesis pathway

**
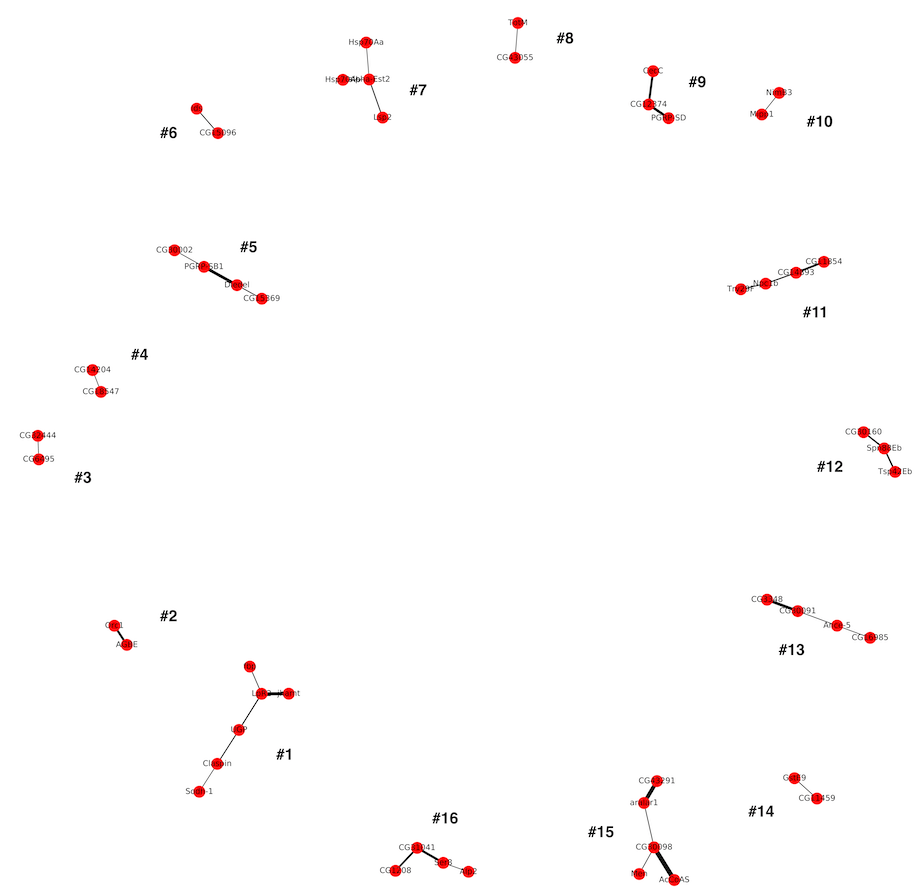
**

**Figure S7. GC filtered network.** The network was pruned to only the edges with at least three consecutive windows of GC significance, those that have a negative GC relationship, and it excludes genes identified with JTK_Cycle.

**
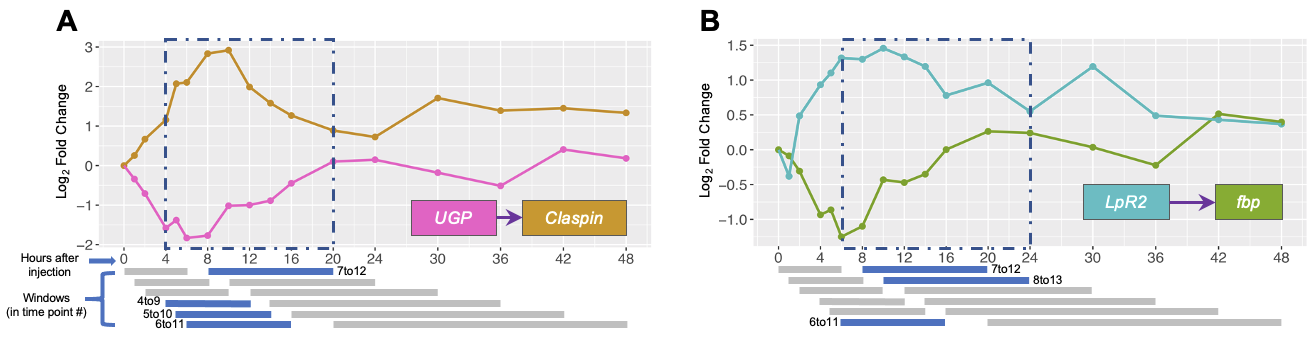
**

**Figure S8. Negative GC edges. (A) between *Claspin* and *UGP.* (B) between *LpR2* and *fbp*** Windows in which a significant Granger Causal relationship is established are colored in blue and are contained in a dashed blue box marking the plot area, non-significant windows are grey below the plot.

**
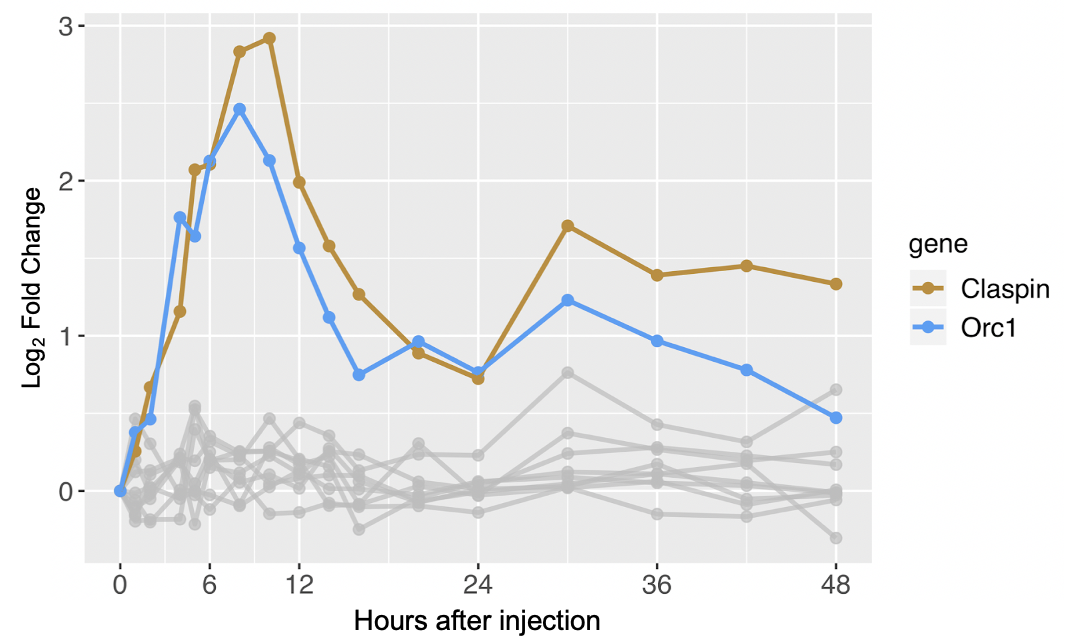
**

**Figure S9. Pathway corresponding to ‘mitotic DNA replication checkpoint’**

**
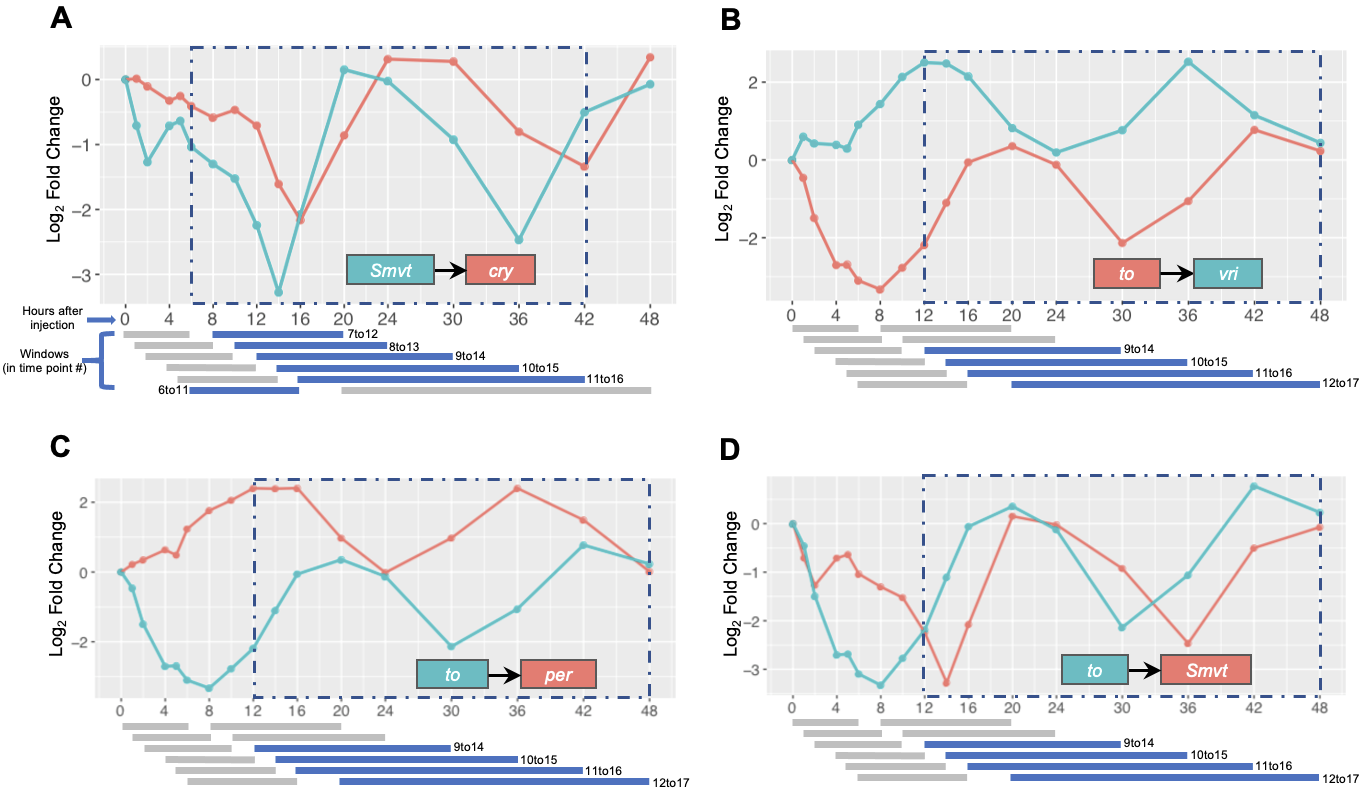
**

**Figure S10. GC edges of circadian rhythm genes plotted against time.** (**A**) Positive lead-lag correlation between *Smvt* and *cry*. (**B**) Negative lead-lag correlation between *to* and *vri*. (**C**) Negative lead-lag correlation between *period* and *takeout.* (**D**) Positive lead-lag correlation between *Smvt* and *takeout*. Significant windows are colored in blue, non-significant windows are colored in grey. Resulting overall consecutive windows are labeled in blue dashed rectangles. Individual windows represent 6 consecutive time points. Note that time points are not regularly distributed with time, therefore windows have different time ranges, but identical number of samples.


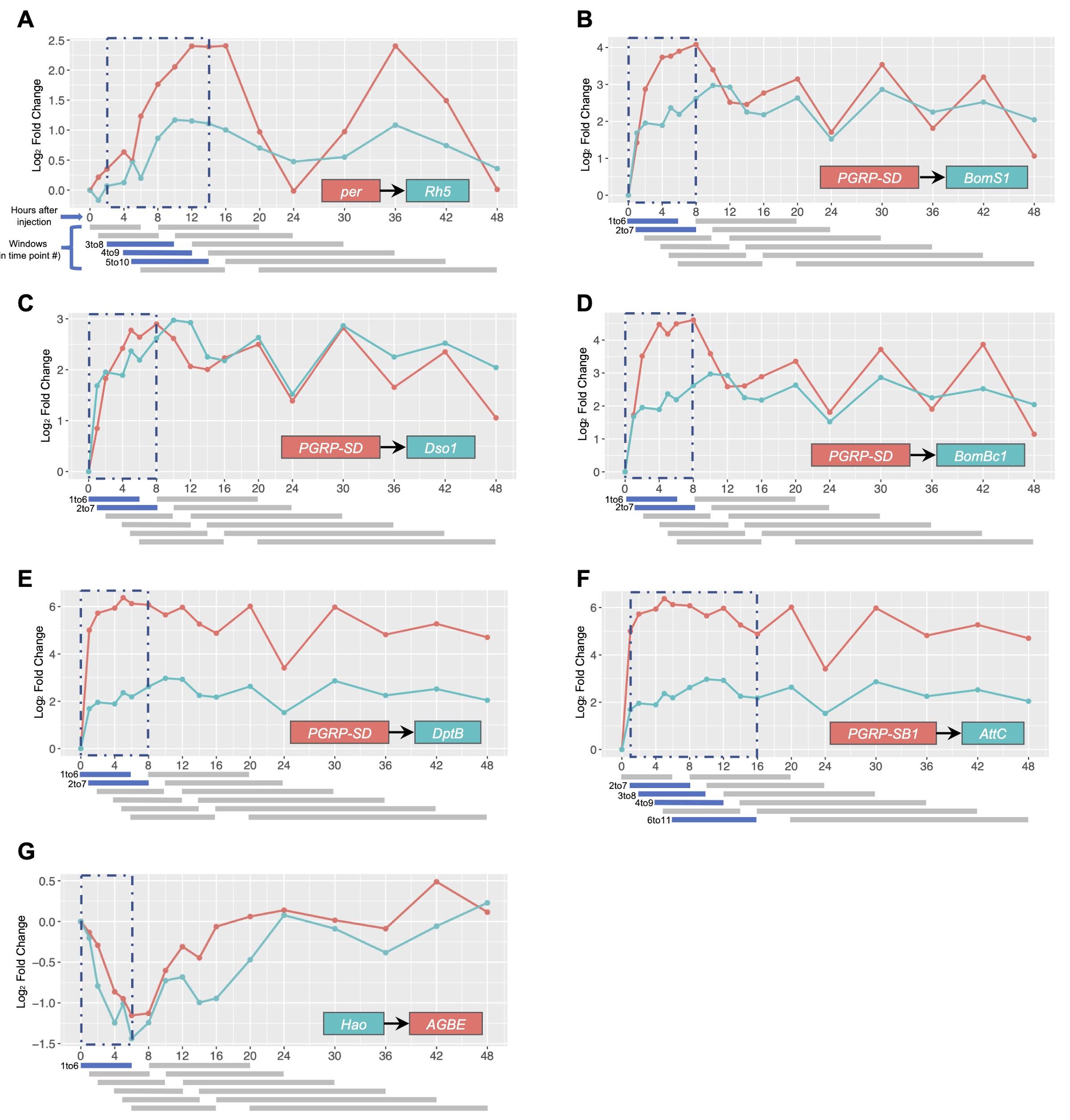


**Figure S11. Positive GC edges.** Positive lead-lag correlation between (**A**) *period* and *Rh5*, (**B**) *BomS1* and *PGRP-SD,* (**C**) *Dso1* and *PGRP-SD,* (**D**) *BomBc1* and *PGRP-SD,* (**E**) *DptB* and *PGRP-SD,* (**F**) *AttC* and *PGRP-SB1,* and (**G**) *AGBE* and *Hao.* Significant windows are colored in blue, non-significant windows are colored in grey. Resulting overall consecutive windows are labeled in blue dashed rectangles. Individual windows represent 6 consecutive time points. Note that time points are not regularly distributed with time, therefore windows have different time ranges, but identical number of samples.


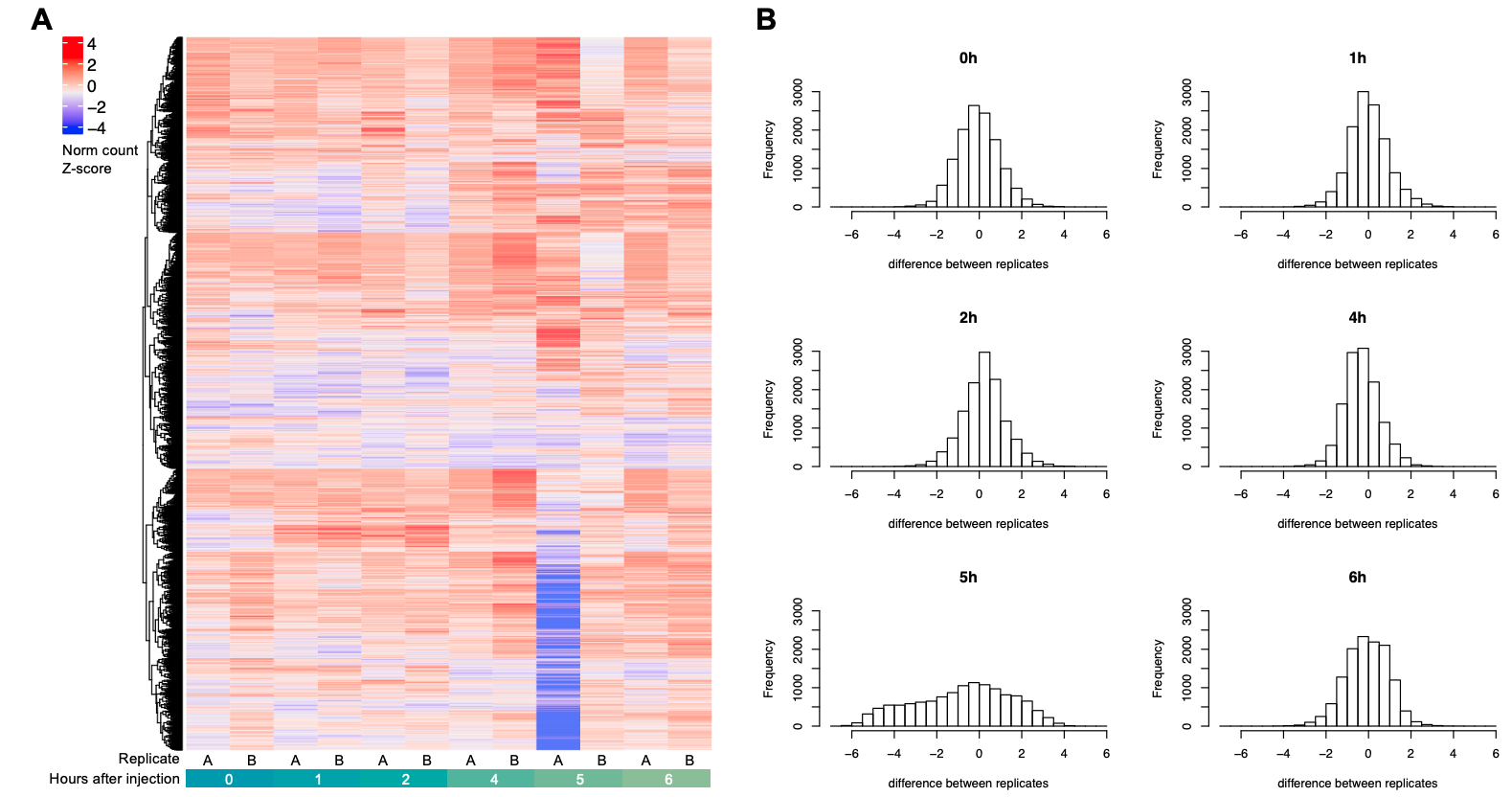


**Figure S12. Sample 6A is an outlier for a subset of genes*.*** (**A**) Heatmap showing row Z scores of normalized counts for all 12,657 genes in the filtered datasets. Each row represents a gene; each column represents a replicate, from pre-injection (0 hours) up to 6 hr post-injection. (**B**) Distribution of differences between normalized count Z scores for replicates A and B for each time point.
