# Supplementary material for: Dense time-course gene expression profiling of the Drosophila melanogaster innate immune response
Source: BMC Genomics. 2021 Apr 26;22:304. doi: 10.1186/s12864-021-07593-3 (PMC8074482; doi:10.1186/s12864-021-07593-3)
Supplement: Supplementary file 3 — Additional file 3 Table S2. Genes that encode transcription factors and respond to commercial LPS injection. [file 12864_2021_7593_MOESM3_ESM.docx]

**Table S2. 20 genes that respond to commercial LPS injection encode transcription factors*.***

| **Symbol** | **Flybase ID** | **Flybase snapshot** |
| --- | --- | --- |
| *E(spl)m3-HLH* | FBgn0002609 | Enhancer of split m3, helix-loop-helix (E(spl)m3-HLH) is a member of the enhancer of split gene (E(spl)) complex. E(spl)m3-HLH encodes a transcriptional repressor executing Notch-mediated cellular differentiation. |
| *GATAe* | FBgn0038391 | GATAe (GATAe) encodes a endoderm-specific GATA factor. It regulates endoderm differentiation and intestinal stem cell maintenance. |
| *CG10147* | FBgn0035702 | NA |
| *exex* | FBgn0041156 | extra-extra (exex) encodes a homeodomain transcription factor that is expressed in and regulates the differentiation of motor neurons that project axons to ventral body wall muscles. Within the central nervous system, the product of exex negatively interacts with the products of Lim3 and eve to govern neuronal specification and differentiation. The roles of the product of exex include neuronal specification and differentiation. |
| *CG2678* | FBgn0014931 | NA |
| *vri* | FBgn0016076 | vrille (vri) encodes a bZIP transcription factor acting as an enhancer of dpp phenotypes both in embryo and in wing. It is involved in hair and cell growth and in tracheal development. Vri is a clock-controlled gene acting as a repressor of the products of Clk and cry. |
| *Pdp1* | FBgn0016694 | PAR-domain protein 1 (Pdp1) encodes a member of the PAR domain bZip family of sequence-specific transcription factors. It regulates gene expression in muscles and in circadian clock neurons. |
| *Clk* | FBgn0023076 | NA |
| *unpg* | FBgn0015561 | NA |
| *cbt* | FBgn0043364 | cabut (cbt) encodes a transcription factor that controls Dpp signaling and is involved in dorsal closure and wing disc morphogenesis. |
| *sr* | FBgn0003499 | stripe (sr) encodes a transcription factor that induces the fate of tendon cells in the embryo as well as in the adult fly. It works upstream of tendon specific genes including Tsp, slow and Lrt. |
| *CrebA* | FBgn0004396 | Cyclic-AMP response element binding protein A (CrebA) encodes a leucine-zipper transcription factor that upregulates genes encoding protein components of the canonical secretory pathway and tissue-specific genes in the salivary gland and epidermis. |
| *Ets21C* | FBgn0005660 | Ets at 21C (Ets21C) encodes a stress-inducible transcription factor that binds specifically to purine-rich DNA motifs. It cooperates with transcription factors acting downstream of the MAP kinase pathways to fine tune gene expression in response to stress, such as infection or oncogene activation. |
| *Dif* | FBgn0011274 | Dorsal-related immunity factor (Dif) encodes a transcription factor that contributes to zygotic function of the Toll pathway, notably the regulation of antimicrobial peptides, but it is not involved in dorsoventral patterning of the embryo. |
| *Rel* | FBgn0014018 | Relish (Rel) encodes a transcription factor and the downstream component of the immune deficiency pathway, which regulates the antibacterial response and other less characterized cellular processes. |
| *Hr38* | FBgn0014859 | Hormone receptor-like in 38 (Hr38) encodes a protein that can heterodimerize with the U adult cuticle and for the proper uptake and storage of glycogen in larvae. |
| *luna* | FBgn0040765 | NA |
| *p53* | FBgn0039044 | p53 (p53) encodes a transcriptional factor required for adaptive responses to genotoxic stress, including cell death, compensatory proliferation and DNA repair. |
| *Hand* | FBgn0032209 | Hand (Hand) encodes a transcription factor that contributes to cardiogenesis, hemopoiesis, and muscle function. |
| *Fer3* | FBgn0037937 | 48 related 3 (Fer3) encodes a basic helix-loop-helix (bHLH) transcription factor expressed in the ventral nerve cord. |
